# Response of Bortezomib Chemotherapy in Hepatic Amyloidosis

**DOI:** 10.1177/2324709618760079

**Published:** 2018-03-07

**Authors:** Syed M. Hasan, Nida N. Ahmed, Zunirah Ahmed, Allan Seibert

**Affiliations:** 1 University of Alabama at Birmingham, Montgomery, AL, USA

**Keywords:** hepatic amyloidosis, amyloidosis, bortezomib

## Abstract

Amyloidosis is a rare disorder with a wide spectrum of presentations and anomalies. It is subdivided into 2 broad categories based on protein deposition; primary and secondary amyloidosis. It can present as a single-organ involvement or as a diffuse infiltrative multi-organ process. Isolated hepatic amyloidosis presentation is a rare phenomenon that develops due to insoluble amyloid deposition in liver. Its clinical presentation is usually vague and ranges from mild hepatomegaly with elevated liver enzymes to acute liver failure and hepatic rupture. Currently, there are scarce data available regarding treatment options for biopsy-proven hepatic amyloidosis. In this review article, we present an interesting case of hepatic amyloidosis and its poor outcome to new molecular targeted chemotherapy. Furthermore, we aim to review current and future diagnostic tools for early detection and advancements in targeted chemotherapeutics options available for hepatic amyloidosis.

## Introduction

Amyloidosis is a rarely encountered disorder with a wide array of appearances and anomalies. It involves amyloid protein deposition in extracellular or intracellular tissues. Immunoglobulin light chain (AL) amyloidosis (previously referred to as primary amyloidosis) and secondary amyloidosis (AA) are the 2 most frequently encountered subtypes of amyloidosis. AL occurs due to plasma cell clonal disorders resulting in abnormal immunoglobulin light chain deposition throughout multiple organ systems.^1^ It is more common in developed countries. In the United States, AL is the most often encountered subtype with approximately 4500 new cases diagnosed each year.^2^ The mean age of diagnosis is 65 years, and it exhibits a slightly greater predominance in men.^3,4^ The AA subtype involves deposition of serum amyloid A proteins. It generally occurs more in developing countries and is associated with chronic inflammatory conditions such as inflammatory bowel disease, rheumatoid arthritis, and osteomyelitis.^5^

The initial presentation of amyloidosis varies and is contingent on the precise organ systems involved and the severity of the disease. Its protean manifestations range from single-organ involvement to diffuse infiltrative disease in multiple organs. While virtually every organ may exhibit amyloid deposition, it is frequently identified in the central and peripheral nervous systems, kidneys, heart, lungs, and skin. Less commonly it may be seen in the gastrointestinal (GI) system. Here, we report a case of AL isolated hepatic amyloidosis and review the literature.

## Case Presentation

A 66-year-old African American woman with hypertension presented to the emergency department complaining of shortness of breath and fluid retention in her abdomen and lower extremities for 2 weeks. She also reported an unintentional weight loss of 40 pounds in the 5 months leading up to her presentation. She denied any fevers, chills, night sweats, chest pain, cough, nausea, vomiting, hematochezia, melena, or hematemesis. On examination she exhibited a heart rate of 120 beats per minute and a regular rhythm. She was also noted to have increased abdominal girth with a positive fluid wave and 2+ pitting edema bilaterally in her lower extremities ascending to her thighs. Laboratory data were significant for aspartate aminotransferase 107 U/L, alanine aminotransferase 30 U/L, and alkaline phosphatase 1091 U/L. Her total bilirubin was 7.2 mg/dL, and her direct bilirubin was 6.2 mg/dL. Her creatinine was 1.04 mg/dL. A computed tomography (CT) scan of the abdomen/pelvis revealed generalized hepatomegaly with heterogeneous liver parenchyma consistent with hepatic steatosis and moderate ascites. A transthoracic echocardiogram showed an ejection fraction greater than 70% with grade I diastolic dysfunction. She underwent a diagnostic and therapeutic paracentesis, which was negative for spontaneous bacterial peritonitis. Endoscopic retrograde cholangiopancreatography did not demonstrate any obstruction. The patient was started on spironolactone and furosemide along with a sodium-restricted diet. However, her clinical condition continued to worsen with recrudescence of her abdominal distension and swelling. She underwent a transjugular liver biopsy, which demonstrated apple green birefringence with Congo red stain consistent with hepatic amyloidosis. Liquid chromatography tandem mass spectrometry was consistent with amyloidosis lambda light chain and alpha heavy chain type amyloid deposition. Plasma cell analysis by flow cytometry showed no definitive evidence of monoclonal plasma cell proliferation. Oncology was consulted and the patient was started on bortezomib 0.7 mg/m^2^ and low-dose dexamethasone. The patient continued to require admissions for recurrent volume overload. She ultimately progressed to multi-organ system dysfunction and failure. She expired less than 2 months after her diagnosis.

## Discussion

Amyloidosis refers to a constellation of diseases caused by deposition of amyloid protein, which impairs normal organ function. The term “amyloid” was first used by Rudolf Virchow in 1853.^1^ Approximately 23 subtypes have been reported and are classified into localized or systemic forms according to deposition patterns of amyloid proteins.^6^ AL, AA,  β_2_-macroglobulin, and familial are the main subtypes of systemic amyloidosis.^7^

The reported incidence of AL amyloidosis is 9 cases per million persons per year.^8^ The epidemiology of amyloidosis remains poorly described due to its rarity and variable presentations. Various factors confound estimations of its incidence including regional genetic variation and local environmental factors, which influence disease pathophysiology.^9^

The presentation of amyloidosis depends on the site and degree of protein deposition. Renal involvement is the most common, followed by other organs including the lungs, skin, peripheral and central nervous systems, and the GI tract.^10^ Several articles describe amyloidosis confined to the GI tract where it most commonly affects the small intestine.^11^ Symptoms often include changes in bowel habits, abdominal pain, unintentional weight loss, as well as upper and lower GI bleeds.^11-13^ Hepatic involvement typically manifests as steatorrhea, jaundice, anorexia, ascites, and splenomegaly.^14^

Both AL and AA amyloidosis may involve hepatic manifestations; however, isolated hepatic amyloidosis is not well characterized in the literature.^10^ It is usually distinguished by amyloid deposition in the liver parenchyma. Due to amyloid accumulation, extensive hepatocyte compression occurs, which may result in hepatocellular atrophy. Presentations may range from mild hepatomegaly and asymptomatic liver enzyme elevations to hepatic failure and spontaneous hepatic rupture.^15^ Unlike the case we report, approximately 80% of patients with hepatic amyloidosis also have renal, cardiac, or neurological involvement.^16^ Other commonly associated clinical findings include proteinuria (88%), elevated alkaline phosphatase levels (86%), abnormal serum protein electrophoresis (64%), and hepatomegaly (81%). The median survival is reported to be 9 months.^15,17^

Radiographic evaluation for hepatic amyloidosis is often equivocal. Ultrasound may show heterogeneous echogenicity in the liver. CT studies may demonstrate hepatomegaly with heterogeneous regions of hypoattenuation and calcifications throughout the parenchyma.^18,19^ Magnetic resonance imaging usually exhibits increased T1 signal in hepatic parenchyma with no changes in T2 signal.^20^ Radiographic studies may reveal certain abnormalities that are neither sensitive nor specific, and tissue biopsy remains the gold standard for a definitive diagnosis.^18,19^

Congo red stain of tissue demonstrating apple-green birefringence under polarized light has long been considered the sine qua non for the diagnosis of amyloid.^21^ If systemic disease is suspected, the patient should undergo further evaluation for hematologic malignancy, cardiomyopathy, and renal failure.^22^ GI-related amyloidosis patients should undergo endoscopic screening if reported to have weight loss and dyspepsia refractory to medical management.^23^

Despite recent advancements in amyloidosis treatment, no regimen directly targets isolated hepatic amyloidosis.^24^ Early studies demonstrate no significant mortality benefit with colchicine, prednisone, melphalan, or dimethyl sulfoxide.^25,26^ Girnius et al showed improvement in hepatic function in two thirds of patients with systemic AL amyloidosis treated with high-dose melphalan and autologous stem cell transplantation.^27^ Another study demonstrated successful sequential liver and stem cell transplantation leading to disease resolution in patients with hepatic failure secondary to primary AL amyloidosis.^28^

Clonal plasma cells in AL amyloidosis utilize proteasomes, and the proteasome inhibitor bortezomib is one potential form of targeted therapy.^29^ Combination therapy with alkylating agents is also under consideration. Two studies demonstrate a higher response rate and prolonged progression-free survival in patients receiving cyclophosphamide/bortezomib/dexamethasone (CyBorD).^30,31^ A recent European collaborative study demonstrated a projected 5-year survival rate of 55% in patients with primary AL amyloidosis treated with CyBorD.^32^ However, a recent case report depicted poor results with CyBorD therapy in 2 cases of hepatic amyloidosis.^33^ A liver transplant patient with hepatic AL-type amyloidosis achieved positive results with concomitant use of bortezomib and dexamethasone therapy. The patient was started on chemotherapy one-and-a-half months after transplant and achieved remission after one course. No clinical evidence of recurrent disease was noted 22 months posttransplant.^34^ Despite some recent suggestions of progress, the prognosis of primary amyloidosis with hepatic involvement remains poor. Studies report AL with liver involvement to have a median survival of 3 months compared with 20 months if no hepatic involvement is present.^35,36^

Our patient presented with a unique form of amyloidosis not routinely encountered. A CT of abdomen/pelvis demonstrated hepatomegaly. A liver biopsy was required to obtain the diagnosis. She subsequently underwent a bone marrow biopsy, which was negative for monoclonal plasma cell proliferation. Further evaluation did not reveal cardiac or renal involvement. No evidence of chronic inflammatory conditions was found. She was diagnosed with isolated hepatic amyloidosis, which is a rare entity. Due to its rarity and poor prognosis, no specific management protocol has been established. After reviewing the literature for off-label protocols, our patient was started on bortezomib and prednisone combination therapy. Unfortunately, the patient developed multi-organ failure and expired. This case highlights the need for additional investigations of potential therapeutic protocols for isolated hepatic amyloidosis.

## Conclusion

Isolated hepatic amyloidosis is a rare presentation with a dismal prognosis and no promising long-term therapies. A molecularly targeted therapy previously approved for the treatment of multiple myeloma, bortezomib has been used alone and in combination with other chemotherapy agents to treat hepatic amyloidosis. The case described above emphasizes the need for additional studies to establish specific treatment protocols and further characterize adverse effects to reduce the mortality and morbidity associated with this terminal illness.
